# Grain yield and quality of wheat are improved through post-flowering foliar application of zinc and 6- benzylaminopurine under water deficit condition

**DOI:** 10.3389/fpls.2022.1068649

**Published:** 2023-01-12

**Authors:** Mohammad Javad Zarea, Nasrin Karimi

**Affiliations:** Department of Agronomy and Crop Breeding, Faculty of Agriculture, Ilam University, Ilam, Iran

**Keywords:** drought stress, Zn, cytokinin, TaCKX, yield, wheat, phytic acid

## Abstract

**Introduction:**

Zinc (Zn) as an essential micronutrient and cytokinin as phytohormone not only regulate plant growth but also play fundamental roles in plant tolerance against drought stress. Understating the function and the role of cytokinin in combined with an essential micronutrient, Zn, could improve the choice of a sustainable strategy for improvement of plant drought stress. The objective of this field research was to determine the effect of post-flowering foliar application of ZnSO4 and 6-benzylaminopurine (6-BAP) on grain yield and quality of winter wheat under water deficit condition.

**Methods:**

Experiments were conducted under filed condition. Drought was imposed by with holding irrigation at the beginning of flowering till the signs of temporary wilting/leaf rolling appeared, after which all plots were irrigated to field capacity. The foliar treatment consisted of (1) foliar application of water, as control treatment; (2) foliar application of 10 g ha-1 6-BAP; (3) Foliar application of 20 g ha-1 6-BAP; (4) Foliar application of 10 g ha-1 6-BAP plus foliar application of 6 kg ha-1 ZnSO4 solution and (5) foliar application of 10 g ha-1 6-BAP plus foliar application of 6 kg ha-1 ZnSO4 solution 2 days before drought imposition. Data were collected on grain and straw yield, yield attributes, harvest index, flag leaf fresh matter and dry matter weight, TaCKX6-D1 expression, phytic acid content in grains, mycorrhiza colonization rate and succinate dehydrogenase (SD) activity.

**Results:**

According to ANOVA, the factor ‘Zn’ significantly affected leaf relative water content (p < 0.001). Relative water content for plants foliar applied with 6-BAP was not statistically significant. Applying Zn increased yield, straw dry weight, and kernel weight relative to plants sprayed with water alone. Increased grain yield due to foliar application of Zn was associated with decrease in cytokinin oxidase/dehydrogenase (TaCKX) and increase in kernel weight. Results showed that the drought stress significantly decreased 1000-grain weight that was accompanied with over-expression of cytokinin oxidase/dehydrogenase (TaCKX). Foliar application of Zn increased the concentration of Zn in grains. The experimental data on the zinc content of grain indicated no significant difference between the 6-BAP at 10 mg L-1 and control treatment. The phytate to Zn molar ratio was significantly affected by foliar applied Zn, but not significantly by applied 6-BAP. In the present study, SD activity of the hyphae of indigenous arbuscular mycorrhizal fungi (IAMF) associated with plant roots was also assayed. Results disclose that SD activity of IAMF was significantly affected by Zn treatments during grain filling stages.

**Discussion:**

In summary, both foliar applied Zn and 6-BAP had the significant effects on all measured parameters in winter wheat. However, spike number, harvest index and mycorrhizal colonization rate were neither significantly affected by Zn nor 6- BAP. Foliar application of Zn at 0.6% (6 kg ha-1) and higher 6-BAP (20 mg L-1 m-2) promoted wheat growth and performances under imposed drought stress condition. Plant that only foliar sprayed with water showed higher level of TaCKX6-D1 expression as compared to Zn treated plants, indicating these plants were more affected by imposed drought relative to those plants treated with Zn. The results of this study provides evidence that a combination of Zn and 6-BAP could be an effective in improvement of drought tolerance of wheat and prevents grain yield from further reduction in terms of quality and quantity due to drought stress.

## 1 Introduction

Although the earth’s climate has never been stable for thousands of years, but current global warming is unique in its speed and in its magnitude. During the last several decades, arid and semi-arid areas have experienced and extended drought. Farmers have been forced to rely on ground water, steadily drawing down the level of the water in the underlying aquifer. In Iran one-third of its wheat fields are irrigated and the rest are rain-fed, making most production reliant on rainfall. In semi-arid areas, most drought events are occurring during the winter wheat reproductive growth stage. Yu et al. (2018) studied the impact of droughts on winter wheat yield in eastern China. They concluded that drought during the flowering and filling stages had a significant negative effect on winter wheat yield. Drought occurring during anthesis has been reported to significantly decline the number of grains up to 50% (Saini and Westgate, 2000). According to Adil et al. (2022), the ZnO nanoparticles applied at the time of sowing had a positive effect on wheat yield under salt stress.

Remobilization of assimilates stored pre-anthesis and current assimilation determine grain formation and development in cereals such as wheat (Kobata et al., 1992; Inoue et al., 2004). Drought stress after anthesis (during grain filling) negatively restricts photosynthesis, leading to decline in contribution of current assimilates to the grain. Under drought conditions, there is a rapid decline in photosynthesis, limiting the contribution of current assimilates to the grain. However, drought occurring during grain filling increases the contribution of stem reserves stored pre-anthesis to grain (Yang et al., 2000). Under suitable conditions, contribution of stem reserves and assimilates derived from current photosynthates in final grain yield is 10% and 70 to 90%, respectively (Yang et al., 2000; Inoue et al., 2004). It must be noted that the photosynthates of flag leaf have an important role in grain filling, estimated 60% of the grain saccharides obtained from photosynthates in the flag leaf (Lei et al., 2022) that can be restricted by drought occurring during grain filling.

Zn is an essential micro-element with various roles in plant physiology, biochemical processes, activity of numerous enzymes, growth and development have been extensively studied (Cakmak, 2008; Cakmak et al., 2010; Rehman and Farooq, 2016; Rehman et al., 2018; Cakmak and Kutman, 2018; Karimi et al., 2021; Kaur and Garg, 2021; Liao et al., 2022; Sadeghizadeh and Zarea, 2022). Recently, the role of Zn in plant defense against herbivores and pathogens also reviewed by Cabot et al. (2019). However, the role and mechanisms by which Zn confers drought tolerance in plants has received less attention. The use of foliar application of mineral Zn has been shown to improve drought resistance in several crops. In a previous study, Ashkiani et al. (2020) pointed out the advantages of foliar application of zinc sulfate on seed oil yield of rapeseed under drought stress conditions. Rahmani et al. (2019) claimed that foliar application of Zn (1.2 kg ha^-1^) significantly reduced the adverse effect of drought on safflower. These authors attributed improved drought tolerance to higher accumulation of carbohydrate and proline. Yavas and Unay (2016) also showed a crucial role of Zn in resistance of wheat to drought stress. A similar observation was made by Pavia et al. (2019), who noted that application of ZnSO_4_ heptahydrate solution through priming (0.4% Zn for 8 h) and foliar application (0.1% Zn) could enhanced photoprotection mechanisms in drought-stressed wheat. Sun et al. (2021) reported that the application of nano-ZnO (100 mg L^-1^) mitigated the adverse effect of imposed drought stress in maize. Maize plants applied with nano-ZnO showed betterbetter stomatal movement, higher net photosynthetic rate and enhanced water use efficiency. Sun et al. (2020) reported that application of nano-ZnO (100 mg L^-1^) activated the antioxidant enzyme system, resulting in increased tolerance to drought stress in in maize. This Similar with the observations of Wang et al. (2020) who found that nano-ZnO-primed wheat seed possessed more effective oxygen scavenging system. Ma et al. (2017) reported that zinc *via* increasing the transcription and activity of reactive oxygen species scavenging enzymes increased the concentration of antioxidant active substances. Improved antioxidant defense mechanisms due to foliar application of zinc at terminal growth have been also reported by Sattar et al. (2022) in drought induced wheat. In addition, it was reported that the foliar application of zinc increased grain yield and quality of wheat under drought stress condition (Sultana et al., 2018).

Actual big problem that is occurring in some countries is micronutrient deficiency. Zinc is involved in so many biochemical reactions in the body; it is a cofactor for numerous enzymes. Phytic acid is a chemical compound naturally found in grains such as cereal grains. That phytic acid serves as the main storage of phosphorus in the seeds. Phytic acid binds the zinc and it is going to create zinc deficiency. In a set of 330 breeding lines of wheat, Wen et al. (2022) reported that phytic acid ranged from 0.90 to 1.72% with a mean of 1.24%.

Cytokinins are plant hormones that have vital roles in various plant functions like root-shoot interactions, nucleic acid metabolism and cell division (Yang et al., 2016). Role of cytokinins in plants is important, particularly under stress (Hare et al., 1997). Recent research has focused on the effect of cytokinins in environmental stressesd improvement. Yang et al. (2016) reported that exogenous cytokinins improved winter wheat yield under heat stress. Liu and Huang (2002) found that heat stress tolerance was improved in creeping bent grass due to cytokinin application. This improvement was reported to be associated with an increase in antioxidant activities and decline in lipid peroxidation. Xu et al. (2011) claimed that treatment of broccoli florets with 6-benzylaminopurine inhibited chlorophyll degradation by decreasing in chlorophyllase levels. Cytokinin content in the grains was found by Yang et al. (2000) to be involved in grain filling percentage from early grain-filling stage till middle grain filling stage. According to Yang et al. (2003), cytokinins could affect the sink size of the grain in rice by mediating cell division in the endosperm. Endogenous cytokinins levels can be regulated by the enzyme so called cytokinin oxidase/dehydrogenase, CKO/CKX. Some studies claimed CKX genes that negatively regulated the levels of cytokinins have been reported to lead to improved crop yield and abiotic stresses tolerances (Arora and Sen, 2022). However, there is some evidences that indicate up regulation of this gene has also caused increased yield in rice.

The changing in cellular gene expression profiles is the major physiological response of plant to the environment stresses (Charfeddine et al., 2015). Cytokinin oxidase/dehydrogenase (CKX) by catalyzing the oxidation of side chain cleavage causes the degradation of cytokinins. This degradation is an irreversible reaction and results in inactivation of hormones. Thus cytokinin level in tissue is regulated by CKX activity. CKX through mediation in cytokinin concentration in tissue can control cytokinin-dependent processes (Schmülling et al., 2003). To date, CKX is found in various crops such as wheat, arabidopsis, maize, soybean and bean. Gao et al. (2019) reported that Zn content in rice seed was significantly affected by CKX expression. These authors reported that root-specific *Os*CKX4-overexpressing plants exhibited increased Zn nutrient and yield traits. Panda et al. (2018) attributed poor grain filling in rice to greater levels of *Os*CKX expression and lower cytokinin content. Li et al. (2018) reported a significant correlation between decreased *Ta*CKX2.2.1-3A expression and grain number. CKX-overexpressing has been shown to provoke drought and salinity tolerance (Werner et al., 2010; Nishiyama et al., 2011). There are several works indicated that cytokinin-deficient plants can better tolerate salt stress than wild type (Cortleven et al., 2019). The CKX genes have been reported to influence yield-related traits in wheat, barley and in rice (Szala et al., 2020).

Although the effectiveness of foliar application of Zn has been based on numerous studies, few studies have evaluated its effectiveness when combined with a plant growth promoting hormones like cytokinin. Therefore this study aimed to better understand how exposed winter wheat to drought stress response to foliar application of Zn in combination with 6-Benzylaminopurine (6-BAP). This current study investigated grain yield and yield components, cytokinin oxidase/dehydrogenase (TaCKX) gene expression, Zn concentration in grain, grain phytic acid: Zn molar ratios, mycorrhizal colonization rate and succinate dehydrogenase activity of mycorrhiza fungi responses in winter wheat to foliar application of Zn and 6-Benzylaminopurine (6-BAP) and their combination as well.

## 2 Materials and methods

### 2.1 Field experimental design

The experiment was conducted in 2021-2022 at a commercial wheat farm which is located in Dehgolan County, Kurdistan Province at 35˚17ʹN, 47 ° 22′E, West Iran. The experimental design was a 2 by 3 factorial combination of two levels of foliar spraying with Zn and 3 levels of foliar application of 6-benzylaminopurine (6-ABP) arranged in a randomized block design with three replicates. The levels of Zn factor were a single foliar spray of Zn as ZnSO_4_ ×7 H_2_O_2_ at 0.5% concentration (v/v) and unsprayed control (0%). The second factor (6-PAB) consisted of three levels of foliar sprays of 6-BAP including 0 mg·L^-1^ as unsprayed control, 10 mg·L^-1^ and 20 mg·L^-1^. Phosphorus from source of triple superphosphate and nitrogen as urea were spread evenly at the rate of 50 kg ha^-1^ and 100 kg N ha^-1^, respectively, to the experimental field before sowing. Two levels of applied Zn, unsprayed control and foliar applied with 0.6% Zn, were designated as Zn0 and Zn0.6, respectively. The plot width was six 1.2-m rows by 6 m in length, attaining population rate of 130 plant m^-2^. The previous crop was alfalfa. Winter wheat (*Triticum* aestivum, cv Pishgam, a bread-making variety) sowing was done in the fall. Winter wheat was grown in the fall (late-October). Wheat plants get established to the four- or five-leaf stage; they went into a dormancy period and then in mid-April started growing; get the stem elongation phase of rapid growth. Heading took place in early June. Harvest was done in mid-July. All plots received the same cultivation and agronomic management.

### 2.2 Drought imposition

**Figure 1:**
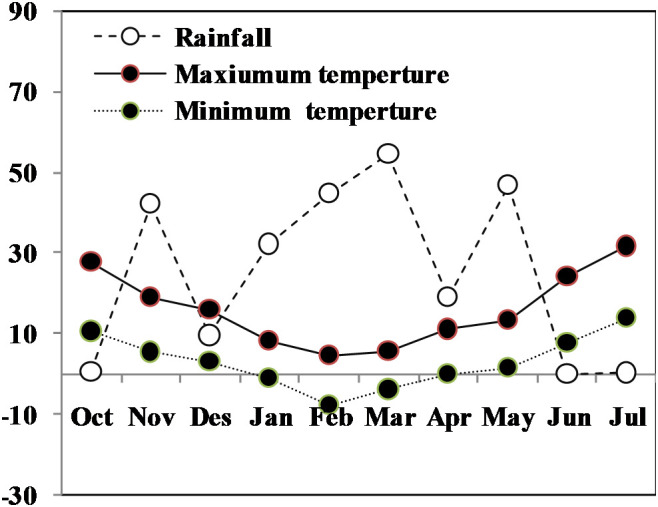
Monthly mean air maximum and minimum temperatures (°C) and rainfall (mm) totals during the whole growing season of winter.

Rainfall and supplemental water included water available for the crop. Monthly rainfall and average temperatures during the wheat growing season in this study is shown in **Figure 1**. Mean rainfall from grain filling to maturity was negligible. Rainfall from drought imposition (heading to maturity) totaled 0.1 mm. Total precipitation from planting to harvesting was 249.6 mm. Drought was imposed by withholding irrigation at the beginning of flowering for 27 days till the signs of temporary wilting/leaf rolling appeared, after which all plots were irrigated to field capacity. Rainfall and supplemental water included water available for the crop. Monthly rainfall and average temperatures during the wheat growing season in this study is shown in **Figure 1**.

### 2.3 Foliar application treatments

The treatment was as follows: (1) foliar application of water (1000 L ha^-1^) as control treatment; (2) Foliar application of 10 g ha^-1^ 6-BAP at a rate of 1000 L ha^-1^ (10 mg L^-1^ m^-2^); (3) Foliar application of 20 g ha^-1^ 6-BAP at a rate of 1000 L ha^-1^ (20 mg L^-1^ m^-2^); (4) Foliar application of 10 g ha^-1^ 6-BAP plus foliar application of 6 kg ha^-1^ ZnSO_4_ as a 0.6% (w/v) ZnSO_4_ 7H_2_O solution at a rate of 1000 L ha^-1^ and (5) foliar application of 10 g ha^-1^ 6-BAP plus foliar application of 6 kg ha^-1^ ZnSO_4_ as a 0.6% (w/v) ZnSO_4_ 7H_2_O solution at a rate of 1000 L ha^-1^ 2 days before drought imposition at 7:00 P.M. Zn and 6-ABP were foliar applied using a hand sprayer with 2-L capacity. Plants were foliar sprayed one time. All solutions included Tween 20 at a final concentration of 0.2% (v/v) as a surfactant. Control plants were also foliar applied with water (1000 L ha^-1^) plus 2 ml L^-1^ Tween 20. The application spray volume was 0.1 L^-1^ m^-2^. To prepare 6-BAP stock solution, 100 mg of 6-BAP was dissolved in water using HCl (1%). 10 and 20 mg L-1 6-BAP were then prepared by diluting the prepared stock solution in water until the volume reaches 1 liter.

### 2.4 Agronomic trait measurements

Data were collected on grain and straw yield, yield attributes (spike number plant^-1^ and kernel number spike^-1^ and 1000-kernel weight), harvest index, flag leaf fresh matter and dry matter weight, whole plant leaf fresh and dry weight and relative water content of flag leaf. To determine grain yield and straw yield, wheat was harvested at the end of spring (mid-July 2022) from a plot of 1 m^2^, after avoiding borders on each side. At harvest, an area of 1 m^2^ of each plot (a total 130 plants) was characterized for grain yield and above ground plant (straw yield). All samples were then dried until constant in weight. Grain yield was represented based on the adjusted moisture content of 120 g kg^-1^. At wheat maturity, aboveground biomass (straw yield) was determined by hand harvesting at ground level from an area of 1 m^2^. Spike number plant^-1^ and kernel number spike^-1^ were determined by counting the spikes and seeds on 3 plants and 3 spikes, respectively at harvest. To determine thousand-grain weight, 3 random spikes per plot were taken from 3 plants and hand shelled. Harvest index (HI) was calculated using the following equation:


(1) $$\begin{array}{c} HI\text{ (}\%)\;=\;\left[grainweight\right]\\ /\left[totaldryweightoftheabovegroundbiomass\right]\;\times \;100 \end{array}$$

Relative water content (RWC) as an important index of water status in plants was measured after drought imposition. RWC cogitate (reflects) the equilibrium (balance) between transpiration rate and water supply to the leaf tissue (Lugojan and Ciulca, 2011). Leaf relative water content was calculated according to the following formula:


(2) $$\begin{array}{c} Relativewatercontent\text{ (\%) }=\;\left[freshweight–dryweight\right]\\ /\left[turgidweight–dryweight\right]\;\times \;100 \end{array}$$

### 2.5 Zn and phytic acid content in grains

To determine the grain Zn concentration, Seed samples were first ground and then were digested in an acid (H_2_O_2_: HNO_3_ at 3:10 v/v ratio). 0.2 g of dried grounded kernel samples was digested using concentrated acid HNO_3_ and H_2_O_2_ mixture. Grain zinc concentration was then determined using a flame atomic absorption spectrophotometer. To measure phytic acid content in grain, the method reported by Liu et al. (2005) with some modification (Lu et al., 2020) was followed. Grain samples from each replicate were dried at 80°C for 48 h and were then grounded to fine powder. Phytic acid assay was conducted according to Liu et al. (2005) with modification. Grain sample (0.4 g) was placed into a 50 ml centrifuge tube and to it 10 ml 0.2 M HCl was added. Tubes were shaken for 2 h. Tubes were centrifuged at 12,000 rpm at room temperature for 10 min. The 2.5 ml of the aqueous phase (supernatant) was transferred into a new tube and to it 2 ml of 0.2% FeCl_3_ solution was added. Samples were boiled in a bath for 30 min. After cooling, tubes were centrifuged again at 13,000 rpm for 15 min. Supernatant was discarded and the tube was washed twice with 5 ml distilled water. Afterwards, 3 ml of 1.5 M NaOH was added into the residue, vortexed for 2 min and then centrifuged at13,000 rpm for 10 min. Supernatant was discarded and 10 ml of 0.5 M HCl was added to dissolve the residue. Finally, deionized water was added to make the volume of 50 m. The indirect method previously described by Haug and Lantzsch (1983) was used to measure the phytate in the extract. Absorbance of the pink color that was developed by 2,20-bi-pyridine with un-reacted Fe^3+^ was recorded at 519 nm. The phytic acid to zinc molar ration was calculated according to the following formula:


(3) $$\begin{array}{c} PhytatetoZnmolarratio\;=\;\left[themoleofphytate\;\left(660.04\;gmol^{-1}\right)\right]\\ /[themolarofZn\;\left(65.4\;gmol^{-1}\right] \end{array}$$

### 2.6 *Ta*CKX6-D1 expression

Expression level of *Ta*CKX6-D1 was determined in grain 7 days after anthesis. Total RNA were isolated from homogenized seed samples. The RNA was isolated using the RNX-Plus buffer. The manufacturer’s instructions were followed for RNA isolation. Grains were first ground in liquid N and immediately transferred to a 2 ml tube, and to it 1 ml of ice cold RNX-Plus solution was immediately added. Shortly afterwards, tubes were vortexed for10 s and, then, were incubated at room temperature for 10 min in horizontal position. 200 µl of chloroform was added to the tubes and mixed thoroughly by shaking for 15s gently. Tubes were incubated on ice for 5 min. Tubes were then centrifuged at 12,000 rpm at 4°C for 15 min. The upper aqueous phase (supernatant) was transferred into a new RNase‐free 1.5 ml tube and equal volume of Isopropanol was added and gently mixed. Tubes were incubated on ice for 15 min. Thereafter, tubes were centrifuged at 12,000 rpm at 4°C for 15 min. The supernatant was discarded and 1 ml of 75% Ethanol was added. Tubes were shortly vortexed to dislodge the pellet and then centrifuged at 4°C for 8 min at 7500 rpm. Supernatant was discarded and the pellet was dried at room temperature for a few minutes. Pellet was dissolved in 50μl of DEPC treated water. To help dissolve, tubes were placed in 55°C water bath for 10 min. The quality and quantity of total RNA were measured by using a NanoDrop. DNA contamination was eliminated by treating total RNA with DNase. cDNA was synthesized using a using Fermentas Nase Kit (Fermentas, Hanover, MD), according to the manufacturer’s instructions and stored at -20°C. Semi-quantitative RT-PCR analysis was performance to evaluate the expression level of *T*aCKX6-D1. The PCR amplification conditions for *Ta*CKX6 gene was as follows: 4 min at 95°C followed by 35 cycles of 30 s at 94°C, 10 s at 55°C and finally 45 s at 72°C, followed by 72°C for 7 min. To optimize the number of PCR cycles for semi-quantitative analysis, PCR products after 20, 25, 30 and 35 cycles were checked for visibility by running on 1.8% agarose gel. The 25 cycle was chosen and quantification was done in comparison to Tacyclophilin gene as reference genes. The primer pairs were designed using CKX6-D1 (Genbank accession: JQ797673). Designed primers were as follows: 5`ATCCATAAGCCTCTCACA ACAGT 3` as forward primer and 5`ACATCG GATCTAGCTTGTTCGT 3`as reverse primer, resulting product size of 170bp. The ΔΔCt method was used to calculate AQP gene expression (Pfaffl, 2001).

### 2.7 Mycorrhiza colonization rate and succinate dehydrogenase activity measurement

After 7 days of drought imposition, root samples were taken from 3 plants of each plot. Root samples were softened using 10% KOH solution. Root samples were boiled in KOH solutions for 15 min. Acidification of softened root samples were done with 1 M HCl solution. After Acidification roots were rinsed in distilled water. 0.02% Trypan blue was used to stain root samples. Root samples were simmering in this solution overnight. Excess stain was removed by 50% lactophenol for 2 h. To determine total root colonization rate, the grid line intersect method of McGonigle et al. (1990) was followed. Hyphae were observed through a grid placed in the microscope eyepiece. To assay succinate dehydrogenase activity (SDA), the method described by Hamel et al. (1990) and Kough et al. (1987) was followed to measure SDA. Roots were collected from three random chosen plants of each plot. Root samples were thoroughly washed with water on a fine sieve. Roots samples were then cut into 1 cm length and placed into 50 ml tub. 20 ml of prepared staining solution was added to the tube. Tubes were incubated at room temperature for 12 h. Staining solution contained 0.05 mM Tris buffer with pH of 7.4. Tris buffer contained Nitro Blue Tetrazolium salt (1 mg ml^-1^), MgCl_2_ (0.5 mM) and sodium succinate (0.25 M). After incubation, stained hyphae were rinsed with distilled water. Roots samples were prepared in polyvinyl alcohol (Hamel et al., 1990). Finally, evaluation of hyphal viability was detected under dark field microscopy.

### 2.8 Data analysis

Recorded data were analyzed (two-way ANOVA) with PROC procedure using SAS software package. When the *F*-test was significant the least significant differences test (*P* = 0.05) was used to determine differences among treatments.

## 3 Results

All plots received the same volume of applied water, cultivation and agronomic management. After that, all plants (all plots) underwent withholding irrigation at the beginning of flowering for 27 days till the signs of temporary wilting/leaf rolling appeared, after which all plots were irrigated to field capacity. The average grain yield of wheat under normal irrigation in selected farms near experimental site was 8.9 t ha^-1^. In the current study average yield across foliar treatments was 4.83 t ha^-1^.

### 3.1 Leaf fresh weight and dry weight

According to ANOVA, the factor ‘Zn’ significantly affected leaf relative water content (*p*< 0.001). Relative water content for plants foliar applied with 6-BAP was not statistically significant. Zn ×6-BAP had not significant effect on leaf relative water content (**Table 1**). After drought stress imposition, the rate of decreasing in relative water content of flag leaf was more in untreated plants than that of plants treated with Zn. Under post-anthesis drought stress, higher relative water content value (72%) was observed in Zn-foliar applied plants (Zn0.6) than those untreated plants (Zn0) (**Table 1**). Plants treated with Zn had a higher relative water content of 11.2% in relation to the untreated plants. The flag leaf fresh weight and dry weight were significantly affected by foliar applied Zn (**Table 1**). The flag leaf fresh weight and dry weight for plants foliar-applied with 6-BAP was not statistically significant. Zn × 6-BAP interaction was not significant for flag leaf fresh weight and dry weight. Plants foliar sprayed with Zn had higher flag leaf fresh weight (0.24 g leaf^-1^) and flag leaf dry weight (0.13 g leaf^-1^) than untreated plants. Results showed that foliar applied Zn resulted in higher flag leaf fresh weight (11.1%) and dry weight (16.7%) in comparison with unsprayed control. Leaf fresh weight and dry weight significantly affected by the factor ‘Zn’. Foliar 6-BAP and Zn × 6-BAP interaction had no significant effect on the above mentioned traits. Leaf fresh weight (3%) and dry weight (7.9%) plant^-1^ were significantly higher for plants sprayed with Zn than that for unsprayed control plants (**Table 1**).

**Table 1:** Effect of foliar applied Zn and 6-benzylaminopurine (6-BAP) on leaf relative water content, flag lea fresh weight and dry weight and total plant leaf fresh weight and dry weight.

Treatment	Relative water content	Flag leaf fresh wt.	Flag leaf dry wt.	Leaf fresh wt.	Leaf dry wt.
	%	---------------g leaf^-1^----------		-------------g plant^-1^------	
Foliar Zn application (%)					
Zn0	65b	0.22b	0.11b	1.21b	0.60b
Zn0.6	72a	0.24a	0.13a	1.25a	0.65a
LSD (0.05)	2.53	0.013	0.012	0.032	0.062
Foliar 6-BAP (mg L^-1^)					
0	68a	0.23a	0.11a	1.22a	0.61a
10	68a	0.24a	0.11a	1.23a	0.62a
20	69a	0.24a	0.12a	1.24a	0.65a
LSD (0.05)	NS	NS	NS	NS	NS
Interaction					
Zn × 6-BAP	NS	NS	NS	NS	NS

NS no significant at 0.05 probability level. Means in a column followed by different lower case letters are significantly different at P < 0.05 according to Fisher’s LSD test.

### 3.2 Yield, straw yield, harvest index and yield components

**Figure 2:**
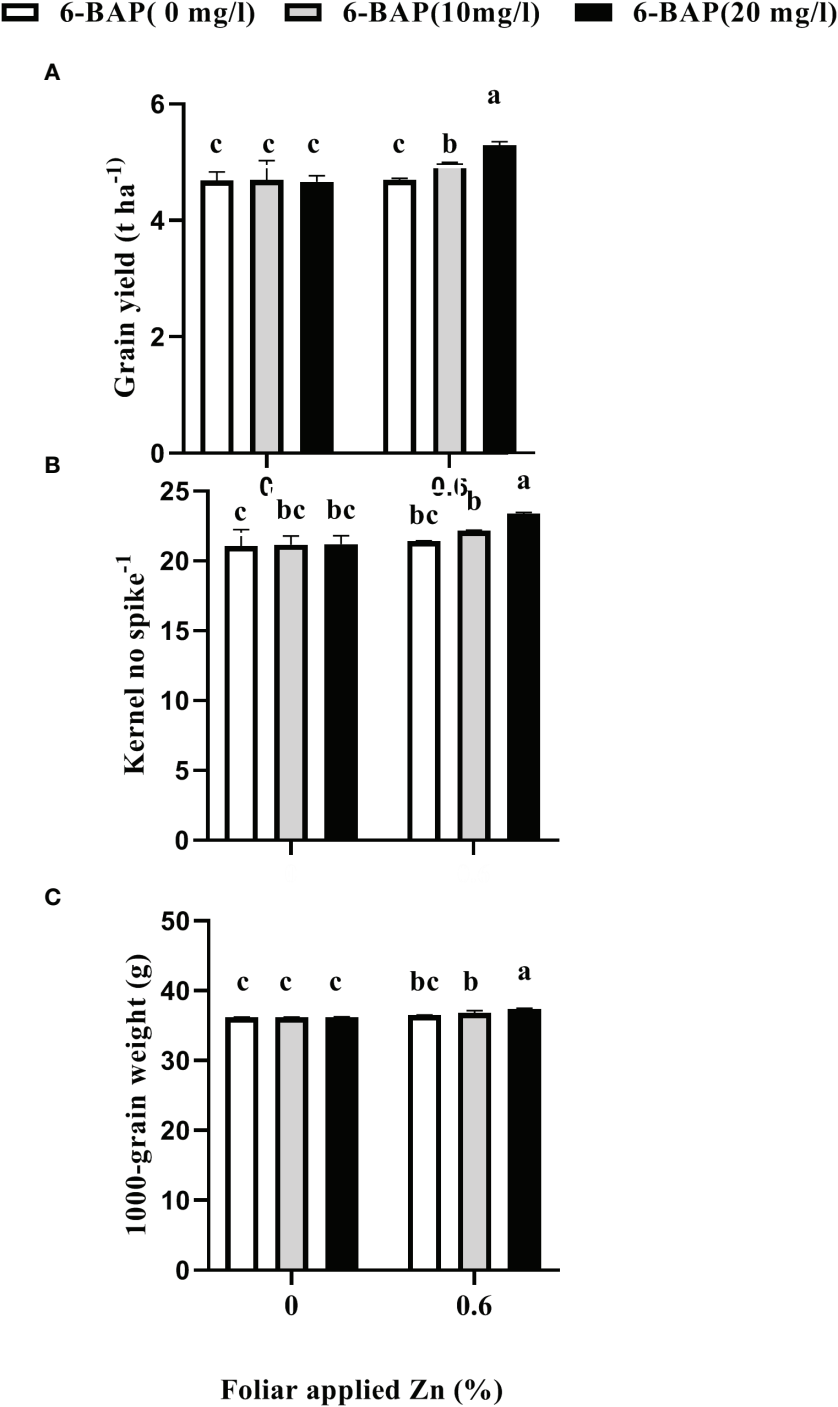
Effect of foliar applied Zn and 6-Benzylaminopurine (6-BAP) no grain yield **(A)**, kernel no spike-1 **(B)**, and 1000-kernel weight **(C)** of winter wheat under drought stress imposition. Different lowercase letters on the plots indicate significant differences (P < 0.05) among treatment means.

As shown in **Table 2**, the result of ANOVA indicated that the treatment Zn and 6-BAP and their interaction were significant for grain yield. Both foliar treatments (Zn and 6-BAP) had significant effect on yield (**Table 3**). As shown in **Table 3**, yield was 0.3 t ha^-1^ (6.58%) higher for Zn treatment (Zn0.6) than for the control (Zn0). Foliar spraying with Zn enhanced grain yield from 4.68 to 4.98 t ha^-^1 (**Table 3**). The grain yield obtained from 6-BAP (20 mg L^-1^) foliar applied plants was higher than that in plants treated with 10 mg l^-1^ 6-BAP and unsprayed control plants (**Table 3**). Foliar applied 6-BAP at 20 mg l^-1^ increased yield up to 2.89 and 6% compared with 6-BAP at 10 mg l^-1^ and unsprayed control, respectively (**Table 3**). The yield response to foliar applied Zn plus 6-BAP was significantly positive (**Figure 2A**). Combination of Zn (0.6%) and 6-BAP at 20 mg l^-1^ resulted in highest grain yield (5.29 t ha^-1^) (**Figure 2A**). According to ANOVA, the main effect of Zn on total dry matter yield (straw dry weight) was significant. The ANOVA for the data for the straw dry yield showed that the main effect of 6-bap and Zn × 6-BAP interactions were not significant. Total straw dry weight was significantly increased by applied Zn by 0.29 t ha^-1^ (**Table 3**). Harvest index (%) and spike number plant^-1^ were not significantly affected by Zn, 6-BAP and their interaction (**Table 2**). Grain number per spike^-1^ was significantly affected by both foliar applied Zn and 6-BAP and their interaction as well (**Table 3**). As shown in **Table 3**, grain number per spike^-1^ of Zn treatment (22.31) was evidently higher than that of the untreated treatment (21.13). Foliar applied Zn increased number of seed spike^-1^ up to 5.5%. Plants sprayed with 20 mg L^-1^ 6-BAP had higher number of grain number per spike^-1^ than that of control treatment (**Table 3**). Foliar sprayed 20 mg L^-1^ 6-BAP increased grain number spike^-1^ up to 2.9% and 5% as compared with 10 mg L^-1^ applied 6-BAP and control treatment, respectively (**Table 3**). Plants foliar sprayed with Zn and treated with 20 mg L^-1^ 6-BAP (Zn + 6-BAP) gave the highest grain number spike^-1^ (**Figure 2B**). A significant grain weight (1000-kernel weight) response was found for foliar applied Zn and 6-BAP (**Table 3**). Thousand-grain weight was increased by 1.9% due to foliar applied Zn (**Table 3**). Foliar applied 20 mg l^-1^ 6-BAP enhanced thousand-grain weight up to 0.76% and 1.2% as compared to applied 6-BAP at 20 mg l^-1^ and unsprayed control, respectively (**Table 3**). There was significant Zn × 6-BAP interaction for grain weight. 1000-grain weight was the highest for plants foliar sprayed with Zn at 2% and 6-BAP at 20 mg l^-1^ (**Figure 2C**).

**Table 2:** Analysis of variance for the effect of foliar applied Zn and 6-benzylaminopurine (6-BAP) on grain yield.

Source	df	Means square	*F*-value	*P* > *F*
Zn	1	0.42782 ^**^	18.65	0.0015
6-BAP	2	0.12277 ^*^	5.35	0.0263
Zn × 6-BAP	2	0.14731 ^*^	6.42	0.0161

*significant at 0.05 probability level.

**significant at 0.05 probability level.

**Table 3:** Main effect of foliar Zn and 6-Benzylaminopurine (6-BAP) on yield, straw dry weight, harvest index, and yield components of winter wheat.

Treatment	Grain yield	Straw dry wt.	Harvest index	Spike	Kernels	Kernels wt.
	-----------T ha^-1^-----------		%	No. plant^-1^	No spike^-1^	Mg 1000-kernel^-1^
Foliar Zn						
Zn0	4.68a	11.66b	40.27a	4.61a	21.13b	36.20b
Zn0.6	4.98b	11.96a	41.2a	4.51a	22.31a	36.89a
LSD (0.05)	0.15	0.28	NS	NS	0.59	0.26
Foliar 6-BAP (mg L^-1^)						
0	4.69b	11.72a	40.1a	4.56a	21.22b	36.34b
10	4.83ab	11.76a	40.59a	4.58a	21.66ab	36.51ab
20	4.97a	11.95a	41.61a	4.53a	22.29a	36.79a
LSD (0.05)	0.19	NS	NS	NS	0.72	0.32
Interaction						
Zn × 6-BAP	✳	NS	NS	NS	✳	✳

✳ significant at 0.05 probability level.

NS no significant at 0.05 probability level. Means in a column followed by different lower case letters are significantly different at P < 0.05 according to Fisher’s LSD test.

### 3.3 Zinc concentration and phytic acid/Zn molar ratio

Grain zinc content was significantly affected by foliar applied Zn and foliar 6-BAP application (**Table 4**). The Zn × 6-BAP interaction effect on kernel Zn concentration was not significant. As shown in **Table 4**, foliar applied 0.6% Zn (Zn0.6) increased kernel Zn concentration by 13.5% over in relation to the untreated plants (Zn0). The Zn concentration in kernel foliar sprayed with 6-BAP at 20 mg L^-1^ was higher than that of control treatment and 6-BAP at 10 mg L^-1^. The experimental data on the zinc content of grain indicated no significant difference between the 6-BAP at 10 mg L^-1^ and control treatment. The phytate to Zn molar ratio was significantly affected by foliar applied Zn (**Table 4**). Plants foliar sprayed with 6% Zn had lower phytate to Zn molar ratio (29.1%) than that of control treatment. The phytate:Zn molar ratio was not significantly affected by foliar applied 6-BAP. There were no significant Zn × 6-BAP interaction effects for phytate to Zn molar ratio (**Table 4**).

**Table 4:** Impact of foliar applied Zn and 6-Benzylaminopurine (6-BAP) on grain Zn concentration and phytic acid to zinc mole ration.

Treatment	Zn concentration	Phytic acid : Zn
	mg kg^-1^	
Foliar Zn (%)		
Zn0	26.5b	3.72a
Zn0.6	30.1a	2.88b
LSD (0.05)	0.86	0.28
Foliar 6-BAP (mg L^-1^)		
0	27.7b	3.51a
10	28.1b	3.26ab
20	29.3a	3.13ab
LSD (0.05)	1.05	NS

NS no significant at 0.05 probability level. Means in a column followed by different lower case letters are significantly different at P < 0.05 according to Fisher’s LSD test.

### 3.4 CKX expression

**Figure 3:**
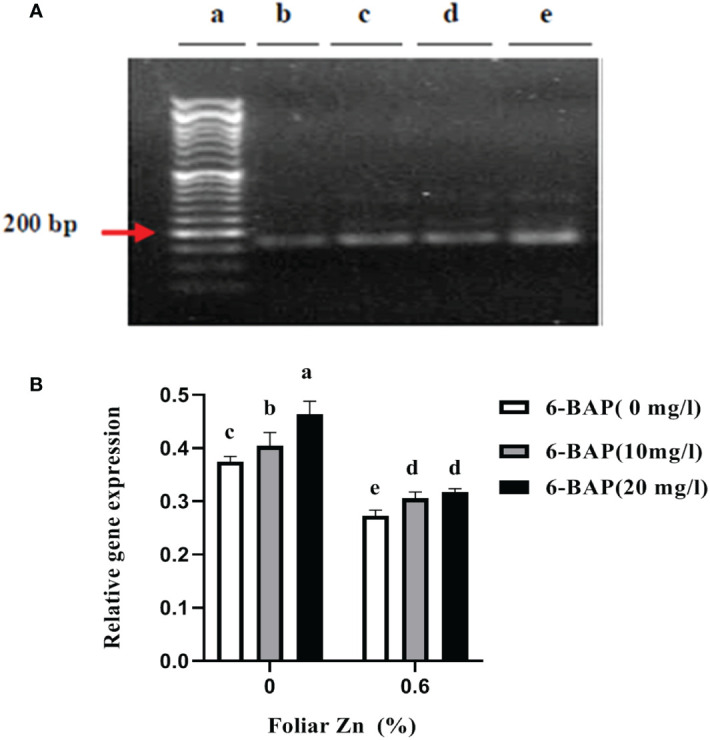
**(A)** Semi quantitative reverse transcription PCR visualization of CKX transcript encoding *Ta*CKX6-D1 expression in winter wheat under drought stress on 1.9% agarose gel. **(B)** Amplification at 25th cycles for transcript encoding *TA*CYTX. (a) Marker; (b) foliar applied Zn + 6-BAP (20 mg L-1); (c) foliar applied Zn + 6-BAP (10 mg L-1); (d) foliar applied Zn + 6-BAP (20 mg L-1) (e) unsprayed control treatment. Different lowercase letters on the plots indicate significant differences (P < 0.05) among treatment means.

**Figure 3A** shows the non-saturated gel image of the reverse transcription PCR ethidium bromide-stained agarose gels for gene expression of CKX. The intensity of the resulting band of RNA transcripts of CKX was visually higher in unsprayed plants. The main effect of foliar applied ZnSO_4_ and 6-BAP significantly affected expression level of CKX in the kernel of winter wheat 5 days after drought stress imposition (**Table 5**). There was a significant effect of Zn + 6-BAP on CKX expression (**Table 5**). Plants foliar applied with Zn showed lower expression of CYTX transcripts compared to those unsprayed plants (**Table 6**). CKX expression transcripts were higher for those plants treated with 6-BAP (**Table 6**). The addition of 6-BAP to the foliar applied Zn, especially at 20 mg L^-1^, resulted in lower expression of CYTX transcripts by 1.4-fold and 1.1-fold compared with following application of foliar 6-BAP (20 mg L^-1^) alone and control treatment, respectively (**Figure 3B**).

**Table 5:** Analysis of variance for the effect of foliar applied Zn and 6-benzylaminopurine (6-BAP) on *Ta*CKX.

Source	df	Means square	*F*-value	*P* > *F*
Zn	1	0.06008	241.92	0.0001
6-BAP	2	0.00672	27.07	0.0001
Zn × 6-BAP	2	0.00105	4.23	0.0466

**Table 6:** Transcript expression analysis of *Ta*CKX6 using semi-quantitative RT-PCR.

Treatment	*Ta*CKX6
Foliar Zn application	
Zn0	0.41a
Zn0.6	0.29b
LSD (0.05)	0.016
Foliar 6-BAP application	
0 mg L^-1^	0.32c
10 mg L^-1^	0.35b
20 mg L^-1^	0.39a
LSD (0.05)	0.02

Means in a column followed by different lower case letters are significantly different at P < 0.05 according to Fisher’s LSD test.

### 3.5 Mycorrhizal colonization and succinate dehydrogenase activity

ANOVA indicated that mycorrhizal colonization percentage was influenced neither by foliar applied Zn nor 6-BAP and their interaction as well (**Table 7**). Succinate dehydrogenase activity (SDA) in roots of plants significantly affected by foliar applied Zn but not to applied 6-BAP (**Table 7**). Zn × 6-BAP interaction was not significant for SDA. Plants foliar applied with Zn exhibited higher SDA compared to untreated plants. At 4 days after drought imposition, plants sprayed with Zn solution performed higher levels of SDA (10.9%) in comparison with unsprayed control (**Table 7**). The effect of foliar applied Zn was not influenced by 6-BAP applied rate.

**Table 7:** Effect of foliar applied on mycorrhizal colonization (MC) percentage and Succinate dehydrogenase activity (SDA).

Treatment	MC	SDA
	%	%
Foliar Zn application (%)		
Zn0	36.2a	52.5b
Zn0.6	34.8a	58.3a
LSD (0.05)	NS	1.93
Foliar 6-BAP application (mg L^-1^)		
0	35.8a	55.1a
10	35.1a	55.2a
20	35.6a	56.0a
LSD (0.05)	NS	NS
Interaction		
Zn × 6-BAP	NS	NS

NS no significant at 0.05 probability level. Means in a column followed by different lower case letters are significantly different at P < 0.05 according to Fisher’s LSD test.

## 4 Discussion

Soil water shortage during grain filling under semi-arid and arid areas is becoming a major constraint in winter wheat production. Drought stress occurring at any stage of crop growth can lead to significant yield losses. However, in wheat, terminal drought is more devastating at reproductive phases (Farooq et al., 2017). Drought stress regularly occurs at the post-anthesis stage and leads to significant reduction in the growth and grain yield of wheat (Ullah et al., 2022). The adaption of a plan to drought stress through nutrition and phytohormones management (foliar application) requires information regarding the effect of nutrient and plant hormone on any particular crop or environment. In the present investigation Zn and 6-BAP were foliar applied before drought imposition. The crop response to exogenous application of 6-ABP and foliar Zn may be dependent on the timing of the foliar application of Zn and phytohormone. We hypothesized that when Zn and 6-BAP being applied before drought stress imposition when plant water content is sufficient, applied Zn and 6-BAP may be better absorbed through stomata rather than Zn and 6-BAP sprayed on drought stressed plants. The results of this study indicated the beneficial effect of foliar application of Zn and 6-ABP on post anthesis drought-stressed wheat yield. However, in the current study, three recorded traits including number of spike plant^-1^, harvest index and mycorrhizal colonization rate were significantly affected neither by applied Zn nor 6-ABP.

In the present study, the number of spike plant^-1^ was similar among all treatments. This could be because the final spike number per plant is mainly determined during vegetative growth stage. On the other hand, foliar treatments at the reproductive growth stage are late for the improvement in the final number of tiller (spike) set. In current study, application of Zn and 6-ABP was made at post-anthesis, when the spikes (tiller) were set. In current investigation, the harvest index was similar in all treatments. Harvest index is defined as the yield of grain divided by the total biological yield (stover + grain). In this study, grain yield was increased only by applied Zn. Similarly, straw yield was also enhanced due to foliar applied Zn. Therefore no significant difference in harvest index was observed among treatments. Mycorrhizal colonization rate was significantly similar in all foliar treatments, indicating non-significant effect of applied Zn and 6-BAP on this trait. This could be because foliar treatment during the grain filling stage is late for the increase in the mycorrhizal colonization rate.

### 4.1 Leaf relative water content and leaf fresh weight and dry weight

In the current study, relative water content of flag leaf and other plant leaves was greater in plants foliar sprayed with Zn. Sun et al. (2021) made similar observations about the improvement of water relations due to applied nano-ZnO. Other work (Sattar et al., 2022) showed a positive effect of Zn as foliar application on leaf water relative content in wheat. Foliar applied 6-ABP did not have a significant effect on the above mentioned recorded traits.

### 4.2 Zn effect on yield and yield components

In cereal such as wheat total grain yield is determined by yield components. In the present study, we observed drought imposed after anthesis markedly declined grain yield of un-treated plants mainly due to less grain weight and number of grains. These results are in agreement with those of Anwar et al. (2021) who reported decreased grain yield in wheat was mainly due to less number of grains and grain weight. Grain yield is determined by its attributes, spike number plant^-1^, grain no spike and kernel weight. Grain number spike^-1^ and grain weight are major components of grain yield in winter wheat that are affected by various environmental factors like drought. Severe drought during anthesis could decrease grain yield up to 50% by reducing the number of grains (Saini and Westgate, 2000). The higher grain yield obtained in this study due to Zn application is associated with improved kernel no and kernel weight, suggesting that applying Zn positively affected these yield components. The results of this study, indicated the beneficial effect of foliar application of Zn on post anthesis drought-stressed wheat yield is in line with other studies reporting positive effects of foliar sprayed with Zn on crops, such as wheat (Yavas and Unay, 2016; Pavia et al., 2019; Anwar et al., 2021; Sattar et al., 2022), pak choi (Brassica rapa L.; Fatemi et al., 2021), safflower (Rahmani et al., 2019). There are investigations that elucidate the role of Zn as foliar spraying or seed priming on drought improvement in plants like wheat. Prior research has determined the adequate supply of Zn improved drought tolerance in wheat, sunflower, tomato, red cabbage and maize (Sun et al., 2020; Umair Hassan et al., 2020; Wang et al., 2020; Sun et al., 2021). Plants foliar sprayed with Zn gave a higher yield of 300 kg ha^-1^ (6.58%) in comparison with control plants. Previous studies had indicated that foliar applying Zn is associated with positive yield response in wheat (Karim et al., 2012). In this study higher yield was associated with improved leaf water content, kernel no. spike^-1^ and kernel weight. There are intensive studies on the positive role of Zn on wheat yield. However, sometimes winter wheat yield response to foliar Zn apparently has no significant effect on wheat yield. According to Wang et al. (2012), foliar application of 0.5% (w/v) ZnSO_4_ H_2_O solution at the beginning of stem elongation and flowering stage had no significantly positive effect on grain yield of wheat. These authors suggested that local soils are not Zn deficient. Karim et al. (2012) reported that, under drought condition, foliar applied Zn increased wheat grain yield up to 19%, whereas foliar applied Zn did not affect grain yield in the absence of drought . Overall, these findings suggest that the positive role of foliar Zn application depends on the soil condition and environmental constraint.

#### 4.3 6-BAP effect on yield and yield components

In the present study, exogenous applied 6-BAP increased grain weight and kernel number per spike. Cytokinin has a stimulatory effect on cell division, grain weight and endosperm cell number (Panda et al., 2018). It has been reported that there is a positive correlation between endosperm cell number with grain filling and grain weigh (Panda et al., 2018). Yang et al. (2016) claimed that treated wheat plants with cytokinins had a positive effect on the number of spikelets on wheat. Similar to wheat, a positive relation between exogenous cytokinin applications with the number of spikelets has been also reported in rice (Panda et al., 2018). Our finding is in line with the finding of Zheng et al. (2016) that increased yield in wheat due to application of 6-BAP is related to increased grain number. However, these authors did not observe any significant effect of foliar application of 6-BAP on 1000-grain weight.

### 4.4 Zn ×6-BAP interaction effect on yield and yield components

In this investigation, straw dry weight, harvest index and number of spike plant^-1^ were not significantly affected by Zn × 6-BAP interaction. However, grain yield and yield components (grain number and grain weight) were significantly influenced by foliar application of Zn × 6-BAP interaction. In current study, grain yield, yield components of grain number and grain weight in untreated plants were significantly lower as compared with plants sprayed with Zn + 6-BAP. The effect of foliar-Zn on grain yield was influenced by 6-BAP application. The increase in grain yield, grain weight and grain number spike^-1^ resulting from foliar applied Zn was dependent on the 6-BAP concentration rate. Grain yield, grain number per spike, and grain weight increased as the concentration rate of 6-BAP was increased from 10 to 20 mg L^-1^. This interaction indicated that grain yield, grain weight and grain number per spike were increased 12%, 11 and 3% by foliar applied 20 mg L^-1^ 6-BAP. Kernel weight and number of kernels per spike are the primary yield attributes of wheat (Holman et al., 2021). Kernel weight is the result of a regulated balance between sink (seed size) and source organ. We hypothesize that the exogenous applied 6-BAP before drought imposition may enhance the sink size (endosperm cell number). In the current study, foliar application of Zn decreased *Ta*CKX6-D1 expression and improved leaf water content that helped in improving yield and yield components of kernel weight and kernel number.

### 4.5 Grain Zn concentration and grain phytic acid:Zn molar ratios

In the present study, foliar Zinc application increased Zn concentration in grain by 13.5% compared to the control (Zn0) treatment. The translocation of foliar-applied Zn can be through trichomes and stomata (Domınguez et al., 2011; Wang et al., 2017). Wang et al. (2017) showed that foliar applied 0.3% Zn resulted in up to 95% increases in grain Zn concentration in winter wheat. Similarly, Yang et al. (2011) and Khampuang et al. (2020) reported that foliar application of Zn at early grain filling improved grain Zn concentration in wheat plants grown in low soil Zn condition. Yang et al. (2011) also found that foliar application of 0.3% of ZnSO_4_ 7H_2_O solution (1.5 kg ha^-1^) at early grain filling resulted in an average increase of 64% Zn concentration compared to the no foliar Zn (control) treatment. In a field experiment conducted in Turkey during the 2003-2004 cropping season, Ozturka et al. (2006) reported that foliar Zn applications (0.68 kg ha^-1^) in the form of ZnSO_4_ 7H_2_O on wheat plants increased grain Zn concentrations. 45% of the total absorbed Zn from the zinc applied to leaves has been reported to translocate from leaf into roots and other parts of shoots under Zn-deficient conditions. In the Zn-adequate plants, this proportion has been reported to be nearly 25% (Haslett et al., 2001; Erenoglu et al., 2002). Phytic acid can chelates micronutrients such as Zn and decrease absorption of it by monogastric animal and human. Humans cannot digest phytic acid because of the absence of enzyme phytase in their digestive tract (Brouns 2021).

To our knowledge, this is the first study that shows a positive effect of foliar-applied cytokinin on grain Zn concentrations. Increased Zn concentration in grain due to 6-BAP may be related to the enhancing of translocation of Zn from vegetative tissues into grain. Foliar-applied 6-BAP may affect translocation of Zn from vegetative tissues into grain. In the present study, foliar applied Zn had a significant effect on the grain phytic acid/Zn molar ratio. Foliar Zn decreased the phytic acid:Zn molar ratio. According to a study reported, foliar applied Zn resulted in reduction of grain phytic acid:Zn molar ratios by 52.0% in winter wheat (Wang et al., 2017).

### 4.6 TaCKX6-D1 expression

Plants involved various mechanisms through by mitigate the adverse effect of drought stress. Modulation in endogenous levels of phytohormones is one of the strategy plant adapted to withstand drought stress. In this study, control plants and foliar 6-BAP treated plants had higher expression of CKX, while plants from Zn treatment had lower rate of CKX gene expression. Endogenous plant hormones are controlled by genes. Endogenous cytokinins levels can be regulated by the enzyme called cytokinin oxidase/dehydrogenase, CKO/CKX. Some studies claimed CKX genes that negatively regulated the levels of cytokinins has been reported to lead to improved crop yield and abiotic stresses tolerances (Arora and Sen, 2022). Therefore higher level expression of CKX gene in control plants might be because these plants were more affected by imposed drought relative to those plants treated with Zn. However, there is some evidences that indicate up regulation of this gene has also caused increased yield in rice. Several investigators (Ma et al., 2017; Sultana et al., 2018; Pavia et al., 2019; Ullah et al., 2019; Ashkiani et al., 2020; Sattar et al., 2022) have studied the role of Zn in improvement of drought tolerance in wheat. Lower CKX gene expression in developing seeds might to because foliar applied Zn could, to some extent, mitigate the adverse effect of the imposed drought. Plants sprayed with 6-BAP, especially with 20 mg L^-1^, had higher expression levels of *Ta*CKX6-D1 in compared to control treatment (unsprayed plants). However, grain weight was higher in these plants sprayed with 6-BAP. We assumed that elevated endogenous concentration of cytokinin due to foliar sprayed 6-BAP had induced *Ta*CKX6-D1 gene expression. Cytokinin oxidase/dehydrogenase regulate the level of local in plant tissue (Li et al., 2018). We postulate that increased expression of CKX in response to exogenous applied 6-BAP is a strategically mechanism to improve plant tolerance to imposed drought. In *Arabidopsis ipt* mutants that had decreased cytokinin exhibited higher drought tolerance in comparison to wild type (Nishiyama et al., 2011). There are numerous studies indicating that cytokinin levels decreased in response to stress. However and in contrast to these investigations, other findings have shown that cytokinine was increased in response to drought stress (Zwack and Rashotte, 2015). Macková et al. (2013) reported that decreased cytokinin levels in tobacco plants were achieved by enhanced gene expression of CKX. Increased kernel weight in plants applied with 6-BAP may be related to prolonged active photosynthesis period during grain filling (Chen et al., 2010). Previous research has shown that cytokinin can regulate senescence and stay-green trait (Li et al., 2018). Yang et al. (2016) reported that exogenous cytokinins through improving stay-green increased the yield of winter wheat cultivars. The expression level of *Ta*CKX6-D1 was lower in plants sprayed with Zn (2% v/v) as compared with unsprayed plants. Plants treated with Zn had higher kernel weight and kernel number per spike. Ashikari et al. (2005) and Panda et al. (2018) reported that there was a negative relation between *Os*CKX2 and grain number in rice. Zhang et al. (2012) also reported an inverse correlation between *Ta*CKX6-D1 and 1000-grain weight in wheat. Szala et al. (2020) observed that grain yield and grain number were positively regulated by TaCKX8 but negatively by *Ta*CKX10. In the present study, plants foliar applied with Zn had higher leaf fresh and dry matter weight and leaf water content, postulating applied Zn could improve drought tolerance. In the present study, foliar Zn+6-BAP resulted in a significant negative increase in CKX gene expression. In the present study, foliar Zn+6-BAP resulted in a significant negative increase in CKX gene expression. On the other hand control plants and foliar 6-BAP treated plants had higher expression of CKX, while plants from Zn treatment had lower rate of CKX gene expression. Some studies claimed CKX genes that negatively regulated the levels of cytokinins have been reported to lead to improved crop yield and abiotic stress tolerances (Arora and Sen, 2022). Therefore higher level expression of CKX gene in control plants might be because these plants were more affected by imposed drought relative to those plants treated with Zn. However, there is some evidence that indicates up regulation of this gene has also caused increased yield in rice.

### 4.7 Mycorrhizal colonization and succinate dehydrogenase activity

The best of knowledge, the potential role of Zn and 6-Benzylaminopurine in improving mycorrhizal colonization and succinate dehydrogenase activity against terminal drought in wheat have never been studied. Neither Zn nor 6-Benzylaminopurine influenced mycorrhizal colonization. This might be because foliar treatment during the reproductive growth stage is late for the increasing in the mycorrhizal colonization rate. At 4 days after drought imposition, plants exogenously foliage applied with Zn solution performed higher levels of succinate dehydrogenase activity (10.9%) in comparison with unsprayed control. The literature search yields no previous research on the highlighting the effects of foliar applications of Zn on mycorrhizal colonization rate and succinate dehydrogenase activity to indicate the finding could occur. Sadeghizadeh and Zarea (2022) reported that applied Zn through seed priming led the succinate dehydrogenase activity of hyphae of *Serendipita indica* to increase in rice root. Amjad et al. (2021) reported that wheat performances in response to arbuscular mycorrhizal fungi (*Glumus intraradisis*) were more profound due to Zn application.

### 4.8 Conclusion

Arid- and semi-arid regions have been severely affected by long term deficit in precipitation. In these areas wheat experiences climate change, hot and dry weather and weather variability, becoming a major threat to winter wheat production. However this situation is not limited to wheat and other crops are negatively affected by this situation. Field research needs to be undertaken to investigate agronomic strategies that lead in alleviation of the adverse effect of drought stress. In summary, both foliar applied Zn and 6-BAP had the significant effects on all measured parameters in winter wheat. However, spike number, harvest index and mycorrhizal colonization rate were neither significantly affected by Zn nor 6-BAP. Foliar application of Zn at 0.6% (6 kg ha^-1^) and higher 6-BAP (20 mg L^-1^ m^-2^) promoted wheat growth and performances under imposed drought stress condition. Plant that only foliar sprayed with water showed higher level of *Ta*CKX6-D1 expression as compared to Zn treated plants, indicating these plants were more affected by imposed drought relative to those plants treated with Zn. Concentrations of Zn and phytates in grain need to be considered as well. This study investigated whether combined foliar application of Zn with cytokinin can improve winter wheat grain yield and quality. Zn (6 kg ha-1) combined with 6-Benzylaminopurine (20 mg L-1) gave the best results in terms of yield and yield quality. The results of this study provides evidence that a combination of Zn and 6-BAP could be an effective in improvement of drought tolerance of wheat and prevents grain yield from further reduction in terms of quality and quantity due to drought stress.

## Data availability statement

The original contributions presented in the study are included in the article/Supplementary Material. Further inquiries can be directed to the corresponding author.

## Author contributions

MZ contributed to the material preparation, performing the experiments, data collection and analysis, validation and writing- original draft preparation; NK assisted in carrying out the field research and data collection contributing. All authors contributed to the article and approved the submitted version.
